# Sonographic Assessment of the Normal and Abnormal Feline Mammary Glands and Axillary and Inguinal Lymph Nodes

**DOI:** 10.1155/2021/9998025

**Published:** 2021-07-02

**Authors:** Nayara S. Moraes, Naida C. Borges

**Affiliations:** Department of Veterinary Medicine, Veterinary School and Animal Science (Escola de Veterinária e Zootecnica-EVZ), Federal University of Goiás (Universidade Federal de Goiás-UFG), Highway Goiânia-Nova Veneza, km 8, Campus Samambaia, Goiânia 74690-900, Goiás, Brazil

## Abstract

Ultrasound has been used as a diagnostic tool in normal mammary glands and mammary tumors of several species. This study aims to describe the B-mode and Doppler ultrasound features of the mammary glands and draining lymph nodes in 32 adult female cats. Group 1 (G1) consisted of 22 cats without changes in the mammary glands. The average age was 45 ± 25.09 months, where 63.6% (*n* = 14) were neutered and 31.8% (*n* = 7) had received progestin at some point for reproductive control. Mammary gland structure was predominantly hypoechoic and homogeneous, with well-defined margins. The average thickness was 1.52 ± 1.59 mm, although it may be affected by estrus, pregnancy, and lactation. In G1, 100% of lymph nodes were homogeneous, 98% were hypoechoic, and 100% were with well-defined margins and hilar vascularization. Group 2 (G2) consisted of 10 cats with mammary nodules. The average age was 88.8 ± 40.5 months, and 70% were intact and all had already received progestin. Ultrasound demonstrated enlarged mammary glands, with nodules of different textures clinically, mainly affecting the abdominal mammary glands (61%). In 33.33%, there were visible mammary ducts. Only 54.17% were homogeneous, 95.83% were hypoechoic, and the margins were regular in 52.08%. Lymph nodes in abnormal mammary chains may present changes in size, shape, echotexture, and echogenicity. Ultrasound examination of the mammary glands and lymph nodes are possible to evaluate the entire mammary chain as well the superficial inguinal and axillary lymph nodes for abnormalities in the feline.

## 1. Introduction

In veterinary medicine, neoplasia has been identified as one of the main causes of pet mortality and occurring predominantly in old age [1–3]. Felines are mainly affected by cutaneous neoplasms, lymphomas, and mammary neoplasms. Mammary neoplasia is the third most frequent cancer in these animals, representing 17% of the total of neoplasms in female cats and 12% of the total cats, when considering both genders [4–8].

The mammary gland most frequently affected by neoplasia in cats has not been determined, and studies are quite divergent [9–11]. Clinically, mammary tumors are firm, nodular, single, or multiple masses [12]. Ultrasound examination of the mammary gland provides information about the normal glandular parenchyma, but this pattern may present echogenicity variations due to hormonal action, such as during pregnancy and lactation [10, 11].

Ultrasound is a standard procedure for breast imaging in women due to rapid technological advancement, and it is indicated for the differentiation of solid and cystic nodules detected by mammography or evaluation of palpable nodules. This is usually the initial examination chosen in these patients due to the absence of ionizing radiation and high breast density in younger women [13–15].

There are few studies of the ultrasound evaluation of feline mammary glands, and this examination is not routinely performed in small animals. Ultrasound of superficial structures such as the mammary ducts and lymph nodes is difficult even when using high-frequency transducers.

This study aims to describe the B-mode and Doppler ultrasound features of the normal mammary glands and regional lymph nodes (superficial axillary and inguinal) and to compare the normal ultrasound characteristics to those in abnormal mammary glands and lymph nodes.

## 2. Materials and Methods

The study was approved by the Animal Use Ethics Committee of Federal University of Goiás (CEUA/UFG) (number 076/17).

Clinical and historical data were collected from 36 female cats including breed, age, body weight, age at the first estrous cycle, administration of progestagins, previous or current pregnancy, clinical examination of the mammary glands by palpation, and visual analysis of the mammary chain. The inclusion criteria were bodyweight over 2 kg, age of at least ten months old, and the cats needed to allow manual restraint during the ultrasound examination. Four cats did not meet these criteria and were excluded. The cats included were evaluated about the estrous cycle phase with empiric criteria, considering the history information about time of the last estrus.

Group 1 (G1) consisted of 22 cats without changes in the mammary chain and group 2 (G2) with 10 adult cats with palpable mammary alterations. In addition to breast chain ultrasound, all cats underwent abdominal and regional lymph node ultrasound. Additional thoracic radiography was performed on G2 cats to investigate possible metastatic lesions.

B-mode and Doppler mode ultrasound of the mammary chains and inguinal and axillary lymph nodes was performed with a SonoScape S6 Portable Ultrasound device (SonoScape Medical Corporation) with a 15 MHz linear frequency for mammary gland evaluation and 7.5 MHz linear frequency transducer for evaluation of the abdomen. Images were obtained in longitudinal and transverse planes and assessed for thickness, echotexture (homogeneous and heterogeneous), echogenicity (hyperechoic, isoechoic, and anechoic), margination (well defined, poorly defined, smooth, and irregular), the presence or absence of ducts, and the presence or absence of vascularization. The mammary gland data were divided into left and right cranial and caudal thoracic and cranial and caudal abdominal glands and assessed individually. Lymph nodes were similarly evaluated for shape (oval, rounded, or elongated), size (length and width), margins, echotexture, echogenicity, and vascularization and were classified as suspicious or not for regional metastatic lesions when relevant.

Axillary lymph nodes can be identified by scanning cranial-lateral cranial thoracic mammary glands (CrTMGs) with the transducer positioned parallel to the long axis of the body (longitudinal plane) with a slight medial inclination of the transducer at approximately 10°. The superficial inguinal lymph nodes of the cats are identifiable in a caudolateral scan to the caudal abdominal mammary glands (CaAMGs) with the transducer positioned in a longitudinal plane with a medial inclination of approximately 10° to 15°. The caudal epigastric artery can be scanned a little deeper and cranially to superficial inguinal lymph nodes. The mammary chains scanning protocol was performed first in the longitudinal planes and later in the transverse planes, starting from the left superficial inguinal lymph node, following to the left caudal epigastric artery, the left (CaAMG), maintaining the cranial direction and the decreasing order of the mammary glands (CaAMG, CrAMG, CaTMG, and CrTMG) to the adjacent axillary lymph node and subsequently performing the same procedure on the right side.

The ultrasound findings of each animal were correlated to age, number of pregnancies, phase of the estrous cycle, reproductive status (intact or neutered), and whether or not the cat had received progestogens.

Radiographs were performed in left lateral, right lateral, and ventrodorsal projections (Philips®, model KL74/20.40-São Paulo, São Paulo, Brazil), and images were scanned by using the FCR CAPSULA (Fujifilm®, model CR IR 357-São Paulo, São Paulo, Brazil).

Statistical analysis was performed by frequency calculations and comparison of means by Student's *t*-test, using Excel software from the Microsoft Office Professional Plus 2016 package.

## 3. Results

Group G1 (*n* = 22) ranged from 10 months to 108 months old (45 ± 25.09 months), and 63.64% (*n* = 14) were neutered, with the average age of ovariohysterectomy 11.76 ± 7.44 months. Among the eight intact cats (36.36%), two were in anestrus, one in estrus, and five in diestrus. Seven (31.82%) of the 22 cats had received progestogens, four of them were intact, and three were neutered at the moment of evaluation. Regardless of progestin use, at least one pregnancy was reported in 22.73% (*n* = 5) of the cats at some point historically, but only one was pregnant at the time of scanning. Twenty cats presented with four pairs of mammary glands, one cat presented with five pairs, and one cat presented with a unilateral supernumerary mammary gland.  Table 1 depicts the measured thickness of each mammary gland. Mammary ducts were only detected in three cats during late pregnancy, estrus, and lactation, respectively (cats *n*° 16, 17, and 18).

**Figure 1:**
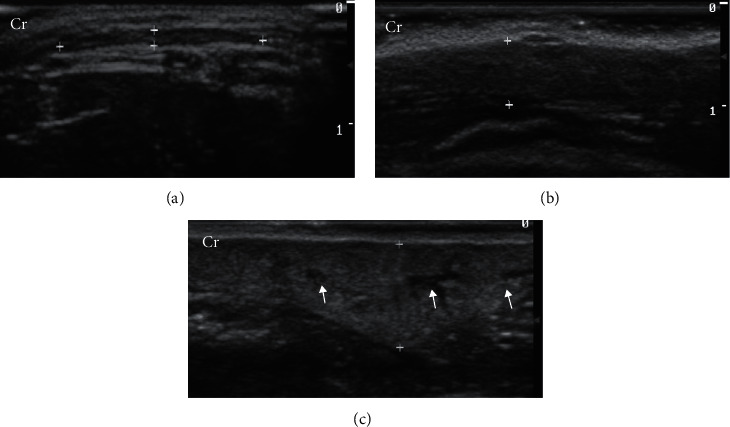
B-mode mammary gland ultrasound (between markers) of healthy cats during anestrus, at late pregnancy, and during lactation. (a) Right caudal thoracic mammary gland with homogeneous echotexture, hypoechoic parenchyma, and well-defined margins. (b) Right caudal abdominal mammary gland with homogeneous echotexture, hypoechoic parenchyma, and less distinct margins. (c) Left caudal abdominal mammary gland with heterogeneous echotexture, slightly hypoechoic parenchyma (comparing to superficial tissue), and the presence of anechogenic mammary ducts (arrows). Cr: cranial. Frequency = 15 MHz.

Mammary glands were classified as homogeneous (95%) and heterogeneous (5%) and as hypoechoic (75%) and isoechoic (25%) (Figure 1). The mammary tissues considered isoechoic were difficult to define as they had subtle margins poorly differentiated from adjacent tissues.

Mammary vascularization was observed with colour flow doppler only during lactation, being characterized by doppler flow, interspersed with the mammary ducts, and an increase in the caliber of a caudal superficial epigastric artery in the region adjacent to the inguinal lymph node.

Group 2 (*n* = 10) was cats with mammary nodules, with ages from 24 months to 144 months (average 88.8 ± 40.5 months). Of the cats, 70% (*n* = 7) were intact, and all (*n* = 10) had received progestogen treatment, with 60% of them receiving regular treatment until the mammary nodules appeared. Previous pregnancy was reported in 50% of the total (*n* = 5) cats. Nine cats had four pairs of mammary glands, and one cat had five pairs of mammary glands.  Table 2 documents the abnormal tissues (thickness). Of all G2 analyzed mammary glands, 31.25% had mammary ducts present.

Palpation demonstrated nodules of different textures, ranging from very soft to very firm and adhering to adjacent tissues. 80% of cats had lesions on more than one mammary gland at the time of diagnosis. 14% of all lesions found were in the cranial thoracic glands (right = 7% and left = 7%), and in 24%, the lesions occurred in the caudal thoracic glands (right = 10% and left = 14%). The involvement of cranial abdominal mammary glands was present in 24% (right = 17% and left = 7%), while the affected caudal abdominal glands were present in 31% (right = 14% and left = 17%). One patient in this group (patient *n*° 8) had a pair of extra mammary gland at inguinal location, also affected by tumor lesions measuring up to 41.8 mm on both sides, making up 6% of the total (right = 3% and left = 3%). Therefore, according to the study conducted, there is a higher probability of tumor involvement in abdominal breasts, which corresponded to 61% of all findings, compared to 38% of findings in thoracic mammary glands.

**Figure 2:**
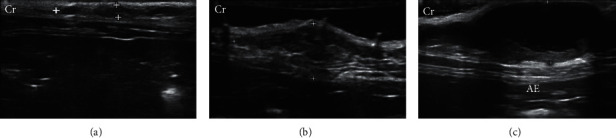
B-mode mammary ultrasound (between markers) of cats with mammary tumors during anestrus. (a) Left caudal abdominal mammary gland with hypoechoic parenchyma, poorly defined margins, and uneven edges. (b) Right cranial abdominal mammary gland with heterogeneous echotexture, uneven shape, unclear edges, and the presence of several lobulations. (c) Left caudal abdominal mammary gland with anechoic content and hypoechoic intratumoral sediment and well-defined margins, creating acoustic enhancement (AE). Cr: cranial. Frequency = 15 MHz.

The mammary ducts were observed in 33.33% by B-mode examination, when considering both groups. Abnormal glands were homogeneous (54.17%) and heterogeneous (45.83%) echotextures, hypoechoic (95.83%), had anechoic (4.17%) appearance and regular margins, well delimited (52.08%) and irregular, and difficult to delimit (47.92%) (Figure 2). Other signs observed were acoustic enhancement (55.56%), posterior acoustic shadow (11.12%), intratumoral sedimentation (38.90%), and intratumoral vacuoles (anechogenic areas between intratumoral tissue) (33.34%). Doppler mode vascularization identified tortuous vessels (70%), peripheral vascularization (20%), hilar-associated peripheral vascularization (30%), intratumor-associated peripheral vascularization (20%), and hilar and intratumoral-associated peripheral vascularization (20%). Only 10% of mammary lesions did not present ultrasound alterations of the vasculature.

**Figure 3:**
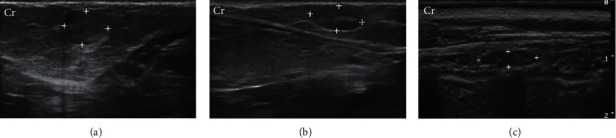
B-mode ultrasound of lymph nodes in a two-year-old cat with abnormal mammary glands and a three-year-old cat with normal mammary glands. (a) Right superficial inguinal lymph node (between the markers) of rounded shape and heterogeneous echotexture, without the central hyperechogenic line. (b) Left superficial inguinal lymph node (between markers) with oval shape, hypoechoic parenchyma, and homogeneous echotexture, with the central hyperechogenic line. (c) Right axillary lymph node (between markers) with elongated oval aspect, homogeneous echotexture, and hypoechoic parenchyma, without central hyperechogenic line. Cr: cranial. Frequency = 15 MHz.

For the G1 lymph nodes (axillary and inguinal), 100% were homogeneous, with well-defined margins and hilar vascularization, with the oval-shaped inguinal and the elongated axillary node, ranging from ovoid to flattened. 65% of lymph nodes had hypoechoic parenchyma with central hyperechogenic marking; in 33%, the parenchyma was hypoechoic without a central echogenic marking, and in 2%, the tissue was isoechoic with central hyperechogenic marking. 57.14% lymph nodes of G2 were classified as oval shaped, 28.57% rounded, and 14.29% were oval shaped, but with easily identifiable lobes. Only 7.50% of G2 lymph nodes had a well-defined central hyperechogenic marking (Figure 3).

Only two cats of G1 had accessory lymph nodes present, meaning that more than one lymph node was draining the mammary chain on either side. One cat had two lymph nodes in the right inguinal region and two lymph nodes in the left inguinal region, and another cat had two lymph nodes in the left axillary drainage. Cats of G2 showed no additional nodes.

The results of the abdominal ultrasound demonstrated changes in 60% of G2 cats and 45.5% of G1 cats. Urinary sediment was the most common ultrasound finding, followed by splenomegaly, hepatomegaly, biliary sludge, renal cysts, uterine dilation, and hydronephrosis. When considering both groups, in 50% of cats, no changes were observed. Heterogeneous nodular masses, possibly secondary to mammary disease, were observed in one animal of G2 in the medial iliac lymph node associated with hepatomegaly and splenomegaly. Radiographic examination of the thorax showed possible pulmonary metastasis in 10% of G2 cats.

In G2, the cats (30%) were neutered after 24 months of age, a period longer than that for the cats of G1, which were neutered at 11.7 months on average.  Table 3 demonstrates the frequency between groups on different analyzed variables as some reproductive aspects, echogenicity, and echotexture of the mammary glands, as the presence of mammary ducts visible by ultrasound examination and lymph nodes sonogram characterization on each group.

## 4. Discussion

Specific knowledge is necessary in order to perform a mammary ultrasound in cats. Early neutered cats have underdeveloped mammary glands that make the examination more difficult to perform. We found that standard ultrasound examination of mammary glands is usually less than 2 mm thick and may increase during estrus, pregnancy, and lactation. This information was similar in the literature [11]. Mammary glands presented a hypoechogenic pattern and homogeneous echotexture during the anestrus and may present mammary ducts during estrus and diestrus during gestation or lactation. According to Payan-Carreira and Martins-Bessa [11], these ducts are responsible for the heterogeneity when the glands undergo hormonal actions, which was confirmed by our findings.

Extra- and supernumerary mammary glands were observed in three cats, and these findings were also described in female dogs [8, 16]. In dogs, there are most commonly five pairs of glands, but supernumerary paired or unpaired glands may occur. Cats usually have four pairs [16].

In the G2, mammary pathology was detected at an average of 7.4 years of age, characterizing earlier involvement than in most published studies, which mention an average of 10 to 14 years of age [3, 12, 17, 18]. In our study, 80% of the cats had nodular involvement in multiple mammary glands, similar to the reviewed literature [1, 9, 19–21].

There was a higher probability of nodular involvement in abdominal mammary glands, corresponding to 62% of all findings, similar to a consulted study that found 62.96% [12]. However, the descriptions of the likelihood of nodular involvement in each gland are controversial [12, 19, 20] requiring further studies.

All cats in the G2 group received progestogens, while only 31.82% of the healthy cats received the contraceptive. Several reversible contraceptive methods have been used in felines, but most protocols used have low efficacy and, especially, low safety [22]. The use of progestogens is related to the appearance of benign and malignant mammary neoplastic lesions [8, 12, 22, 23], due to simultaneous exposure of the mammary tissue to endogenous estrogen and progesterone with exogenous progestins, increasing progestin-induced diseases, then being contraindicated for cats since the risk of disease should be avoided [22]. Although, female cats have a reduced expression of estrogen receptors in neoplastic mammary tissue, with loss of hormonal dependence during malignant progression, explaining, in part, the high biological aggressiveness of malignant mammary tumors [19].

Only 30% of the G2 cats underwent ovariohysterectomy (OHE). In these cats, there was no remaining ovary visible on the ultrasound examination, and the cats were neutered after two years of age which is more compared to the G1 group. OHE in cats when performed before one year is capable of reducing the risk of developing mammary gland cancer by up to 86% compared to nonovariohysterectomized cats [8, 17, 23, 24].

Two of the cats evaluated in G2 had distant nodules at the time of the study, and both had multiple mammary glands affected. One patient with a primary thoracic mammary nodule had a miliary interstitial lung pattern identified by a thorax radiographic study. However, the result of a normal radiographic examination is not synonymous with the absence of possibly metastatic lesions [1, 7, 25], as chest radiographs are only able to detect pulmonary nodules larger than 4-5 mm [1]. Abdominal ultrasound may also show changes in the structure and morphology of the abdominal organs [7, 26]. Another patient with primary nodules in the abdominal mammary gland had secondary nodules in the abdomen, characterized by splenic and hepatic nodules and development of several other intra-abdominal nodules. These lesions were presumed metastatic in the absence of histopathological sampling. It is estimated that 50% to 70% of cats with malignant mammary neoplasms without signs of metastasis at clinical evaluation will still develop metastatic lesions [1, 7, 19, 25]. In addition, there are other imaging methods much more sensitive to detect small metastatic lesions as computed tomography [26–32], magnetic resonance imaging [26, 29], and scintigraphy [1, 25].

The presence of accessory lymph nodes in cats has been described in the literature, and they can present from 1 to 4 lymph nodes performing axillary and inguinal drainage of mammary glands [33, 34]. In the ultrasound examination, changes in lymph node architecture may be suggestive of neoplastic involvement, especially when there is loss of central hyperechogenic hilus visibility and rounding [35–37]. These characteristics were present, respectively, in 92.5% and 28.57% of the lymph nodes evaluated in G2, but the conclusive diagnosis should be made through cytological and histopathological studies of these lymph nodes, and further research will be necessary to study this relationship.

## 5. Conclusions

Using longitudinal and transverse planes in the breasts and both lymph nodes are possible to evaluate the entire mammary chain, superficial inguinal lymph nodes, and axillary lymph nodes in the B-mode ultrasound examination. The ultrasound examination of the normal mammary chain is less than 2 mm thick and may increase during estrus, pregnancy, and lactation. The patterns of normal mammary gland images are usually hypoechoic and homogeneous echotexture, with defined and regular margins, and may have mammary ducts during estrus and diestrus when in the gestational or lactation phase. In abnormal mammary chains, the gland increases in volume width and may lose definition and regularity of the margins, with more frequent mammary ducts, and may be hypoechoic or anechoic, of homogeneous or heterogeneous texture. Lymph nodes in tumor-affected mammary chains may present changes in size, shape, echotexture, and echogenicity. In Doppler mode, caudal superficial epigastric artery and hilar lymphatic vascularization in normal mammary chains are identified, and in tumor-bearing cats, peripheral neovascularization or mixed patterns in a mammary tumor and lymph node tissue are identified.

## Figures and Tables

**Table 1:** Individual values, average (*X*), and standard deviations (SDs) related to age and mammary thickness assessed by ultrasonography in cats with normal mammary glands (G1).

Cats	Age (months)	Mammary thickness (mm)							
		Thoracic mammary gland				Abdominal mammary gland			
		Cranial		Caudal		Cranial		Caudal	
		Right	Left	Right	Left	Right	Left	Right	Left
1	24	0.9	1.1	0.7	1.2	1.0	1.0	1.3	1.3
2	48	0.9	1.3	0.7	0.7	1.2	1.4	1.5	1.1
3	60	1.8	1.8	0.9	1.3	1.1	1.1	0.8	0.9
4	48	0.5	1.2	0.9	1.0	1.1	0.9	1.9	1.3
5	36	1.4	1.3	0.9	1.1	0.8	1.3	1.0	1.8
6	66	0.7	0.8	1.7	1.1	0.6	1.5	0.7	0.4
7	96	0.9	0.9	0.5	0.6	0.4	0.9	0.8	0.3
8	24	1.2	0.6	0.8	0.7	1.1	1.1	0.9	1.2
9	108	1.1	0.9	0.7	1.1	0.8	0.9	1.5	0.7
10	48	1.3	1.6	1.5	0.7	0.9	0.9	1.2	1.2
11	36	0.7	0.4	1.1	1.6	1.0	0.6	1.1	1.3
12	60	0.4	0.6	1.0	0.6	0.8	0.6	1.3	0.9
13	54	0.6	0.5	0.6	0.5	0.5	0.7	0.8	0.8
14	60	1.5	1.5	1.4	2.0	0.9	1.0	0.5	0.8
15	48	1.3	0.9	1.0	1.2	1.2	1.7	1.1	0.7
16	24	1.1	1.1	1.6	1.3	2.6	3.5	6.1	3.9
17	12	1.5	1.6	2.3	2.2	3.4	2.3	4.7	4.2
18	24	7.2	6.2	3.9	6.0	9.0	6.2	10.3	11.1
19	10	0.5	0.8	0.6	0.5	0.4	0.8	1.5	1.0
20	48	0.9	1.0	1.3	1.7	1.3	1.3	2.0	1.9
21	12	0.7	1.3	1.1	1.3	1.9	1.1	1.9	1.3
22	36	0.9	1.1	0.9	0.9	1.0	1.0	1.2	1.0
*X*	**45**	**1.3**	**1.3**	**1.2**	**1.3**	**1.5**	**1.4**	**2.0**	**1.8**
SD	**25**	**1.3**	**11.1**	**0.7**	**1.1**	**1.8**	**1.2**	**2.2**	**2.3**

**Table 2:** Individual values, average (*X*), and standard deviation (SD) related to age and mammary thickness assessed by ultrasonography in cats with mammary abnormalities (G2) based on palpable pathology.

Cats	Age (months)	Mammary thickness (mm)							
		Thoracic mammary gland				Abdominal mammary gland			
		Cranial		Caudal		Cranial		Caudal	
		Right	Left	Right	Left	Right	Left	Right	Left
1	84	**45.4**	1.3	0.9	**4.9**	**8.4**	1.9	3.2	**14.9**
2	60	1.3	1.2	1.5	1.7	2.2	1.9	**2.7**	2.3
3	144	1.5	**2.2**	1.1	0.9	**2.3**	1.0	1.2	**3.4**
4	132	**7.0**	**3.0**	**8.0**	**6.0**	**13.0**	**39.0**	**51.0**	**28.0**
5	60	1.9	2.1	2.8	**15.0**	**12.0**	**4.0**	**9.0**	**17.0**
6	48	0.9	1.1	**31.0**	2.2	**4.2**	**5.0**	**3.5**	**3.9**
7	24	1.4	1.2	3.0	**9.0**	**3.5**	**100.0**	**4.5**	**6.0**
8	108	1.8	1.5	1.9	2.3	2.1	2.2	2.8	**12.6**
9	96	**4.8**	**4.0**	2.8	3.1	**3.4**	3.2	**12.6**	3.7
10	132	2.2	1.5	**7.2**	2.8	2.1	2.3	2.5	2.7
*X*	88.8	6.8	1.9	6.0	4.8	5.3	16.0	9.3	9.5
SD	40.5	13.7	0.9	9.1	4.3	4.2	31.6	15.1	8.5

^*∗*^Highlighted values are those with palpable nodules.

**Table 3:** Relative frequencies (%) of the reproductive and ultrasound features (mammary glands, mammary ducts, and lymph nodes) in cats without (G1, *n* = 22) and with (G2, *n* = 10) mammary tumors.

Features	G1 (%)	G2 (%)
Reproductive		
Neutered	63.6	30.0
Progestogens	31.2	100.0
Pregnancy	22.7	50.0

Mammary glands		
Homogeneous	95.0	54.2
Heterogeneous	5.0	45.8
Hypoechoic	75.0	95.8
Isoechoic	25.0	0
Anechoic	0	4.2

Mammary ducts		
Present	10.0	31.3
Absent	90.0	68.8

Mammary vascularization		
Normal vascularization	95.5	10
Hypervascularization	4.5	90

Lymph nodes		
Oval	100.0	57.1
Rounded	0	28.6
Lobed	0	14.3
Homogeneous	100.0	85.7
Heterogeneous	0	14.3
Hypoechoic	98.0	85.7
Hyperechoic	0	14.3
Isoechoic	2.0	0
Hyperechogenic central line	67.0	7.5
Regular margin	100.0	85.7
Irregular margin	0	14.3
Normal vascularization	100.0	85.7
Hypervascularization	0	14.3

## Data Availability

The raw data used to support the findings of this study are available from the author upon request.
